# Design and validation of an inertial measurement unit (IMU)-based sensor for capturing camera movement in the operating room

**DOI:** 10.1016/j.ohx.2021.e00179

**Published:** 2021-02-16

**Authors:** Tomas J. Saun, Teodor P. Grantcharov

**Affiliations:** a International Centre for Surgical Safety, St. Michael’s Hospital - Li Ka Shing International Healthcare Education Centre, Canada; b Division of Plastic and Reconstructive Surgery, Department of Surgery, University of Toronto, ON, Canada; c Division of General Surgery, Department of Surgery, St. Michael’s Hospital, Toronto, ON, Canada

**Keywords:** Video recording, Instructional film and video, Wearable electronic devices, Micro-electrical-mechanical systems, Calibration, Motion, Surgeons

## Abstract

Intraoperative surgical video enables better surgical training, continued performance enhancement for surgeons and system-level quality improvement initiatives, however the capture of high-quality intraoperative video of open surgical procedures is difficult. Wearable cameras, typically in the form of a head-mounted action camera are frequently used for this purpose, although the video from these devices often contains significant motion artifact due to movement of the surgeon’s head. When trying to compare the performance of various wearable cameras in the surgical setting, we could not find a motion sensor appropriate for this purpose. We therefore describe in this article the design, assembly and validation of a small sensor that can be attached to wearable cameras in the operating room to objectively quantify camera motion. The sensor incorporates an inertial measurement unit coupled to a microcontroller. Concurrent validity is established by comparing the positional sensing of the device to a geared tripod head that allows for fine, measured manipulations of the sensor in three orthogonal axes. The methodology of capturing, processing and reporting camera movement for a surgical procedure is also detailed.

## Graphical abstract

**Figure:**
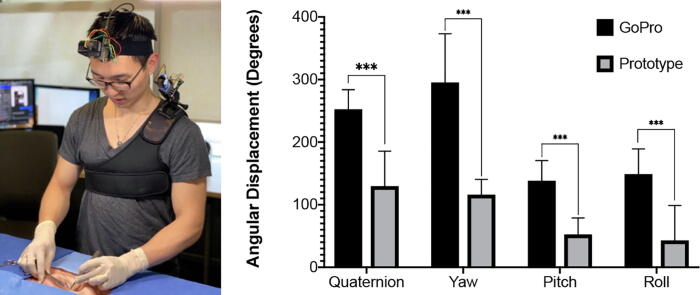

## 1 Hardware in context

High quality intraoperative video capture facilitates better healthcare in many ways, including better surgical training, continued professional development, error analysis and quality improvement. The technology for video recording open surgery, however, is limited. We conducted a systematic review of the literature in 2019 to better understand what solutions were being used for intraoperative video capture. [1] The most commonly cited camera system was the GoPro positioned in a head-mounted configuration. A major limitation of this system, however, is the amount of motion transmitted to the video from the surgeon’s head movements, which makes the captured video difficult to follow. Major limitations of overhead light-handle integrated cameras included obstruction by members of the surgical team and limitations of external arm-mounted cameras were that they required additional personnel and were difficult to appropriately position. Our research group went on to develop an intelligent, stabilized wearable camera device that specifically addressed and overcame many of the limitations of previously described devices.

In the process of innovating this new device, an important goal of our research team was to benchmark the device against previously existing solutions. Unfortunately, there was a paucity in the literature for objective, validated metrics for this purpose. Much of the existing literature simply presented an innovation with no user evaluation reported or used Likert-type surveys with stems developed solely by the authors based on their expert opinion alone. [2], [3], [4], [5], [6] We therefore developed a methodology for both subjective and objective evaluation of these camera devices, specifically for their performance in capturing intraoperative surgical video. The details of our subjective framework have recently been published separately. [7]

One of the key metrics in our objective evaluation was the physical amount of camera movement throughout a surgical case. The performance metric was chosen specifically to evaluate the excessive camera movement seen in head-mounted systems.

The solutions explored for positional tracking of various wearable camera systems can be broadly categorized into two types of approaches: outside in and inside out.[8] Outside in tracking typically utilizes optical sensors placed in the environment surrounding the object to be tracked, which itself is identified with a series of beacons. While this type of system achieves accurate and stable positional tracking, the required setup, specifically the placement of surrounding optical sensors would not be feasible in the operating room environment. Inside out tracking typically utilizes an Inertial Measurement Unit (IMU)-based sensor affixed to the object of interest. While the sensors of an IMU, namely the gyroscope, accelerometer, and magnetometer, individually are insufficient for positional tracking due to factors such as drift and interference, when correctly combined they are capable of very accurate and stable positional orientation tracking.

Off-the-shelf IMU sensors were explored. The issues associated with these products included: cost, size and weight. Furthermore, many of the commercially available sensors relied on additional data sources such as GPS and/or cellular signal for accurate positional tracking, both of which may not be available in the OR setting. Our shoulder-mounted camera device uses a gimbal mount for stabilization and therefore the sensor had to be small and lightweight-enough to not interfere with the balance of the gimbal. We also required software customization so that we could capture the appropriate data output for the comparisons we intended to make.

**Fig. 1:**
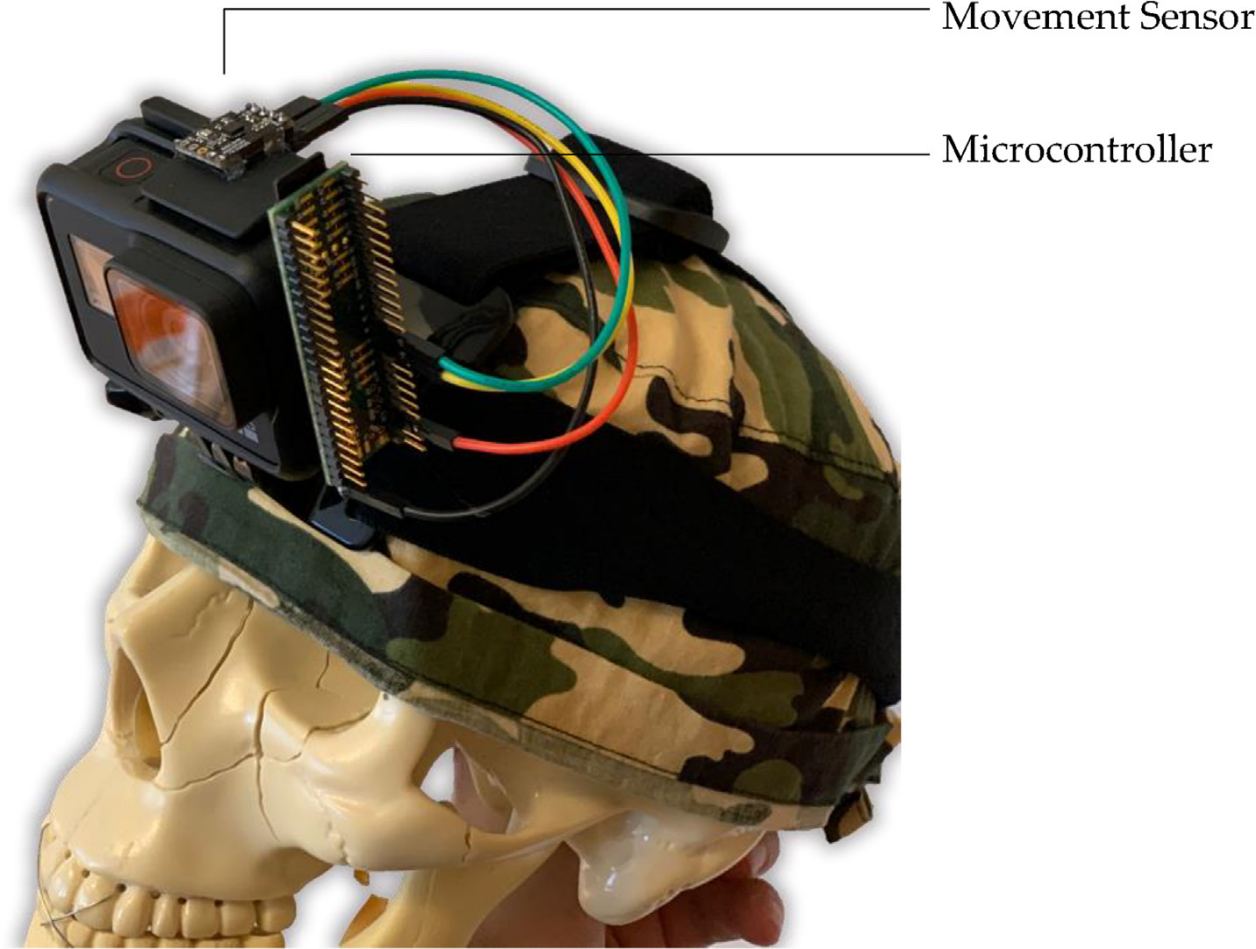
**Surgical Camera Movement Sensor.** Shown is the Surgical Camera Movement Sensor mounted on a head-mounted GoPro camera.

Finding no other suitable hardware, we have developed our own. The surgical camera movement sensor, shown in Fig. 1, is a small and lightweight platform designed to be mounted on a wearable surgical camera to quantify motion. It was designed to be inexpensive to produce, customizable, easily programmable and the design leverages off-the-shelf hobbyist electronics (Table 1). This article, along with the associated project repository, will describe design, basic function, and validation of this sensor.

**Table 1:** Specifications table.

Hardware name	Surgical camera motion sensor
Subject area	• Medical
Hardware type	• Measuring physical properties and in-lab sensors • Field measurements and sensors
Open Source License	GNU General Public License v3
Cost of Hardware	$121.00 CAD
Source File Repository	https://doi.org/10.17605/OSF.IO/9XGH6

## 2 Hardware description

### 2.1 Sensors

Inertial Measurement Units (IMUs) are small electronic devices consisting of some combination of gyroscopes, accelerometers and magnetometers. Once processed through a fusion algorithm, these IMU sensors can accurately measure the orientation of a device in 3D space.

Sensor fusion can broadly be categorized as software vs. hardware fusion. In software fusion, the motion sensor data is fed to a separate microcontroller that then executes fusion algorithms, such as the Kalman, Madgwick or Mahony algorithms. [9] Hardware fusion involves embedding a dedicated processor along with the motion sensors such that the fusion algorithms are executed on the embedded processor, the outputs of which are then provided to a separate microcontroller. Hardware fusion off-loads sophisticated filtering and autocalibration onto an optimized processor resulting in better response and accuracy.

An IMU with hardware fusion was selected to measure positional data. The sensor needed to be as small and lightweight as possible. It also required some degree of accuracy and needed to be easy to use.

**Fig. 2:**
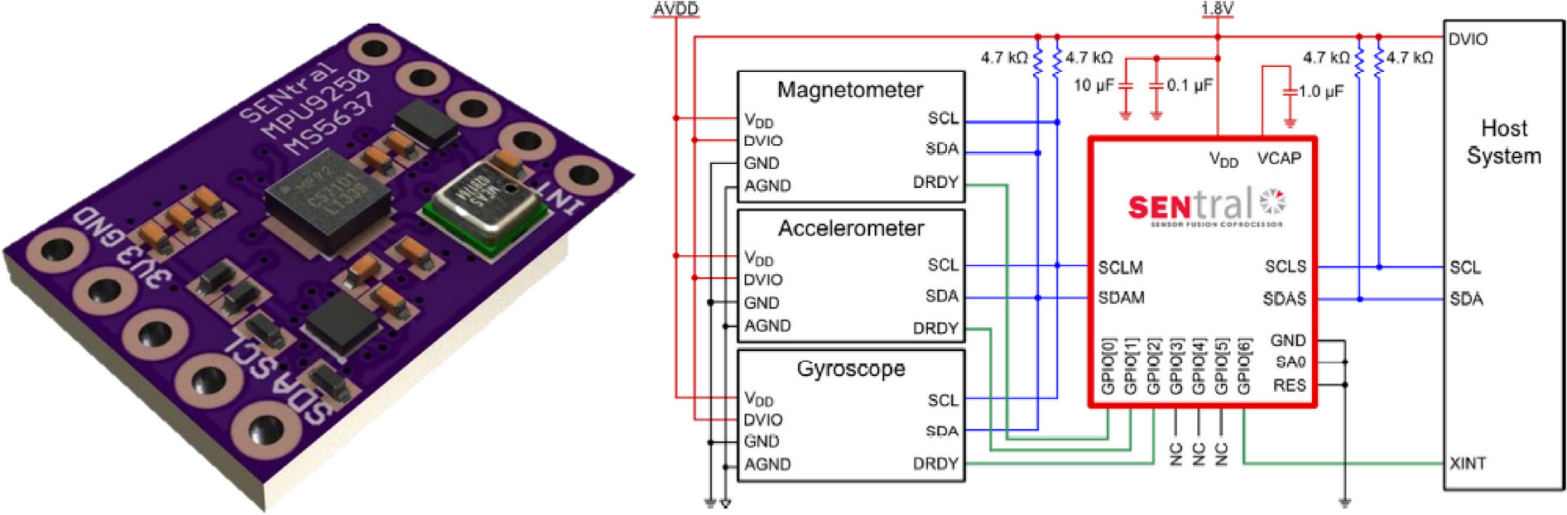
**Ultimate Sensor Fusion Solution** (designed by Kris Winer of PeskyProducts, ThelaCorp). Left: CAD model, Right: circuit diagram.

The Ultimate Sensor Fusion Solution was selected as it met the above criteria and provided extensive support documentation. This sensor integrates the MPU9250 IMU (InvenSense, TDK Corp.), the M24512 I2C EEPROM (STMicroelectronics N.V.), and the EM7180 sensor hub (EM Microelectronic-Marin SA). The MPU9250 IMU is a nine-axis microelectromechanic system (MEMS) motion sensor with embedded accelerometers, gyroscopes and magnetometers. The 64Kbyte M24512 I2C EEPROM stores the sensor configuration file and warm start parameters, which allows for faster initialization times by saving previous initialization and calibration parameters. The EM7180 is a sensor fusion hub (or motion sensor co-processor) takes sensor data from a slave accelerometer, gyroscope, and magnetometer and fuses them. This additional processor allows for better sensor data provided by the MPU9250, excellent dynamic calibration and filtering algorithms, and higher processing speeds (Fig. 2).

### 2.2 Microcontroller

**Fig. 3:**
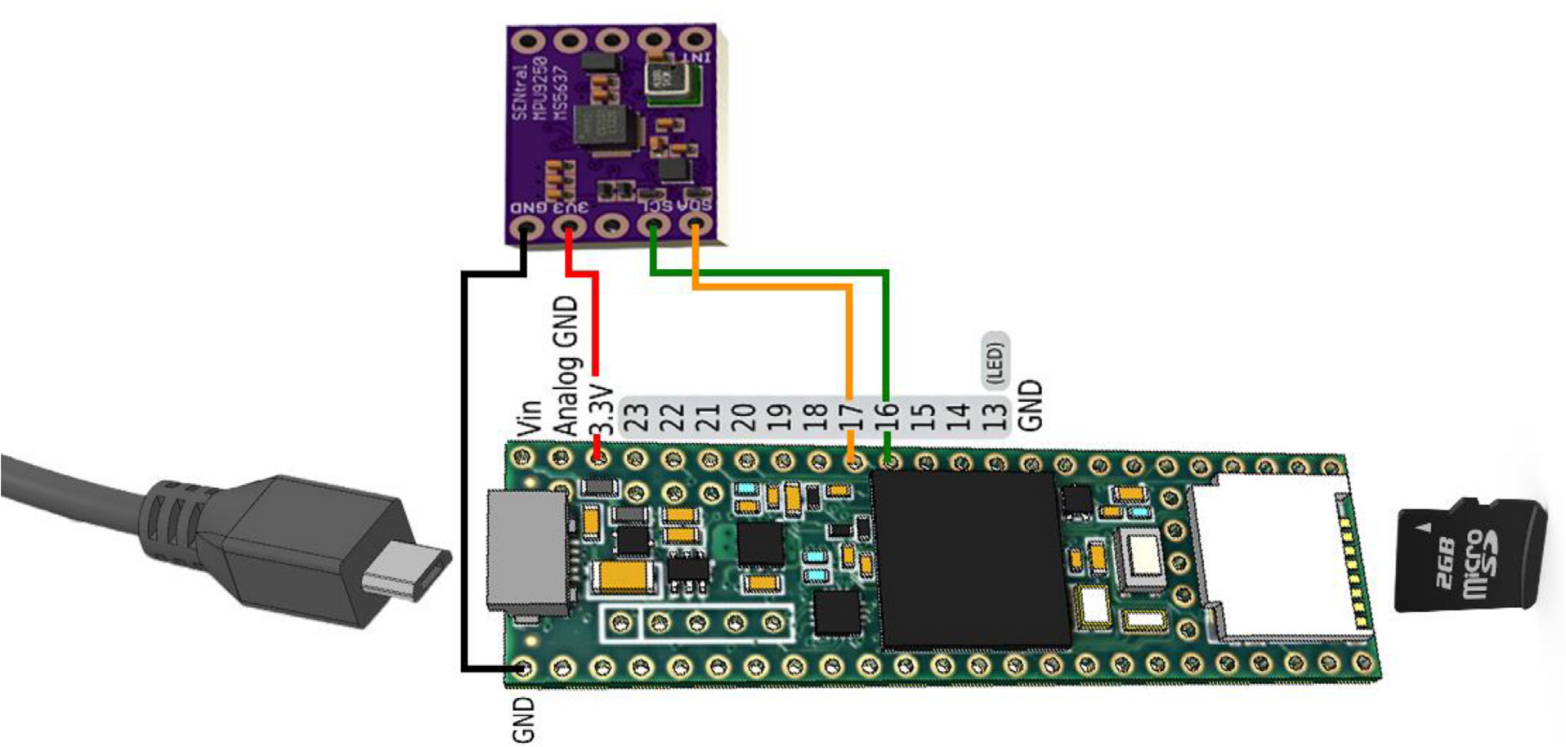
**Global Surgical Camera Movement Sensor Assembly.** Shown is the Ultimate Sensor Fusion Solution (top) coupled to a Teensy 3.5 microdevelopment board (bottom) with attached USB cable (left) and SD card (right).

The sensor was coupled to a Teensy 3.5 microdevelopment board, containing a 120 MHz Cortex-M4F processor and USB and SD card interfaces (PJRC.COM, LLC). This was powered with a short USB-micro cable attached to a USB power bank. The entire assembly is detailed in Fig. 3.

### 2.3 Software

The microcontroller was programmed using the Arduino Integrated Development Environment (IDE). The configuration files were provided open-source from the sensor developer. These were modified to serve the particular functions needed for our project. Sketches were loaded with the microUSB cable and then stored in the onboard memory of the microcontroller.

Important features of the provided code, aside from basic functionality, included functions for autocalibration and then subsequent warm-start capabilities. Accelerometer and magnetometer data can be influenced by non-ideality and environmental factors, and calibration involves moving the sensor in a controlled fashion for a short period of time to ‘teach’ the algorithm to converge to the correct directional estimates. Warm start capability involves loading previously stored calibration data from the EEPROM so as to maintain stable calibration parameters. It is also important to set the appropriate magnetic declination for your location in the source code for accurate heading determination.

Specific software modifications were made for the purposes of this project. The LED indicator on the microcontroller board was set to turn on once the sensor initialization was completed to indicate active sensor data acquisition and storage. The data sampling rate was arbitrarily set to 10 Hz. Data logging to a microSD card was enabled to allow for untethered data acquisition. The data output type was set to both Euler angles (yaw, pitch, roll) as well as quaternions.

### 2.4 Processing

Quaternions are the preferred mathematical number system for calculations involving three-dimensional rotations, however Euler angle representations in the form of yaw, pitch, and roll angles are often reported as they are conceptually more straightforward.

[10] The IMU sensor uses a proprietary, integrated adaptive algorithm to determine quaternions. These quaternions are then used to construct a 3x3 rotation matrix and the yaw, pitch, and roll Euler angles can then be constructed from the direction cosine elements of the matrix (Equations 1 and 2).

$$a12=2.0f*\left(q\left[1\right]*q\left[2\right]+q\left[0\right]*q\left[3\right]\right);$$

$$a22=q\left[0\right]*q\left[0\right]+q\left[1\right]*q\left[1\right]-q\left[2\right]*q\left[2\right]-q\left[3\right]*q\left[3\right];$$

$$a31=2.0f*\left(q\left[0\right]*q\left[1\right]+q\left[2\right]*q\left[3\right]\right);$$

$$a32=2.0f*\left(q\left[1\right]*q\left[3\right]-q\left[0\right]*q\left[2\right]\right);$$

$$a33=q\left[0\right]*q\left[0\right]-q\left[1\right]*q\left[1\right]-q\left[2\right]*q\left[2\right]+q\left[3\right]*q\left[3\right];$$

**Equation 1:** Constructing the rotation matrix from quaternion values

Elements of the 3x3 rotation matrix are calculated from quaternion values

$$\mathrm{pitch}=-asin\left(a32\right);$$

$$\mathrm{roll}=atan\left(a31,a33\right);$$

$$\mathrm{yaw}=atan\left(a12,a22\right);$$

**Equation 2:** Calculating Euler angles.

Euler angle are calculated from the direction cosine elements of the rotation matrix.

What is being described:

• A small and lightweight positional sensor
• A process for validating a positional sensor
• A method of comparing positional changes or motion of wearable sensors
• An experimental methodology and results that use this sensor to objectively quantify and compare motion of two different wearable cameras for intraoperative video recording of surgical procedures

## 3 Design files

All design files have a GNU GPL v3 open source license

Design files can be found at: https://doi.org/10.17605/OSF.IO/9XGH6 (Table 2)

**Table 2:** Design files.

**Design file name**	**File type**	**Description**
*global_schematic.png*	*Schematic*	*Surgical Camera Motion Sensor global schematic*
*sensor_circuit.png*	*Circuit diagram*	*Ultimate Sensor Fusion Solution circuit diagram*
*sensor_CAD.png*	*CAD model*	*Ultimate Sensor Fusion Solution CAD rendering*
*sample_photo.png*	Photo	*Assembled sensor mounted on go-pro*

## 4 Bill of materials

(See Table 3)

**Table 3:** Bill of Materials.

**Component**	**Description**	**Cost [$CAD]**	**Source of materials**
Ultimate Sensor Fusion Solution	Integrates the MPU9250 IMU (InvenSense, TDK Corp.), the M24512 I2C EEPROM (STMicroelectronics N.V.), and the EM7180 sensor hub (EM Microelectronic-Marin SA).	50.00	Pesky Products, USA
Teensy 3.5 microdevelopment board	120 MHz Cortex-M4F processor and USB and SD card interfaces (PJRC.COM, LLC), with header pins soldered in place	39.00	Creatron Inc
Male Pin Headers	90-degree male pin headers (for Ultimate Sensor Fusion Solution)	1.00	Creatron Inc
Female Pin Headers	1 Pin JR (F) Connector Set of 2 ($0.50 × 4)	2.00	Creatron Inc
Wiring	Flexible 30AWG silicone wire	2.00	Creatron Inc
Adhesives	Scotch Extreme Fasteners	*5.00*	Staples Canada Inc
USB cable	USB(A) to Micro(B) Cable – 5ft	6.00	Creatron Inc
Power bank	Anker PowerCore + Mini 3350mAh lipstick-sized portable charger	16.00	Amazon.ca
	**TOTAL**	**121.00**	

## 5 Build instructions

i) Assemble lead wires. Identify the minimum distance from sensor to microcontroller and cut 4 individual lead wires. We used 30AWG silicone wire to ensure lightweight and flexible wiring. Each wire had a single pin receptacle socket attached to either end, effectively creating a short female-to-female lead wire.
ii) Solder 90-degree male pin headers to Ultimate Sensor Fusion Solution on the side with the SDA, SCL, 3V3, and GND terminals.
iii) Attach adhesive backing to sensor and microcontroller. After trialing several types of adhesives we decided to use Scotch Extreme Fasteners, a heavy-duty plastic hook and loop-type adhesive. This allowed for easy mounting and removal of the components and fixed the sensor in a rigid position with respect to the camera device which ensured accurate positional determination.
iv) Connect lead wires between sensor and microcontroller as shown in Fig. 3.
v) Mount circuit boards on camera device as best suited for the specific device. Fig. 1 shows one of our use case applications on a head-mounted GoPro device.

## 6 Operation instructions

### 6.1 Initial setup

Ensure that the hardware is built and connected as specified above. For first time use, the microcontroller must be connected to a computer using a microUSB cable to load the code onto the EEPROM of the microcontroller. This was done using the Teensyduino add-on for Arduino IDE. While connected to the computer, sensor initialization can be viewed on the Arduino IDE console including the keypress option to undergo calibration. Once calibration has been completed, the calibration parameters will be stored on the device. Upon subsequent device initializations, the lack of user input (keypress) defaults to warm start parameters and automatically begins data logging. This allows for device functionality when untethered from the computer.

### 6.2 Data capture

For data capture, we opted for an untethered solution so that the surgeon could move freely. To do this, we enabled SD card logging as described above. For a power supply, we used a generic USB power bank that could be placed in the surgeon’s pocket. This was attached to the microcontroller using a short USB cable.

Once the USB cable was plugged into the power bank, the device would initialize and automatically begin data capture and logging. The flashing LED indicator shows that the device has finished initialization and is actively capturing and logging data. The reset button on the microcontroller could also be pressed to terminate and re-initialize the device.

### 6.3 Data transfer

Data is stored as comma separated values (CSV) in a text file on the microSD card. This can be transferred to a computer using a standard SD card reader. The CSV data can be parsed into Excel or MATLAB for further processing and analysis.

## 7 Validation

**Fig. 4:**
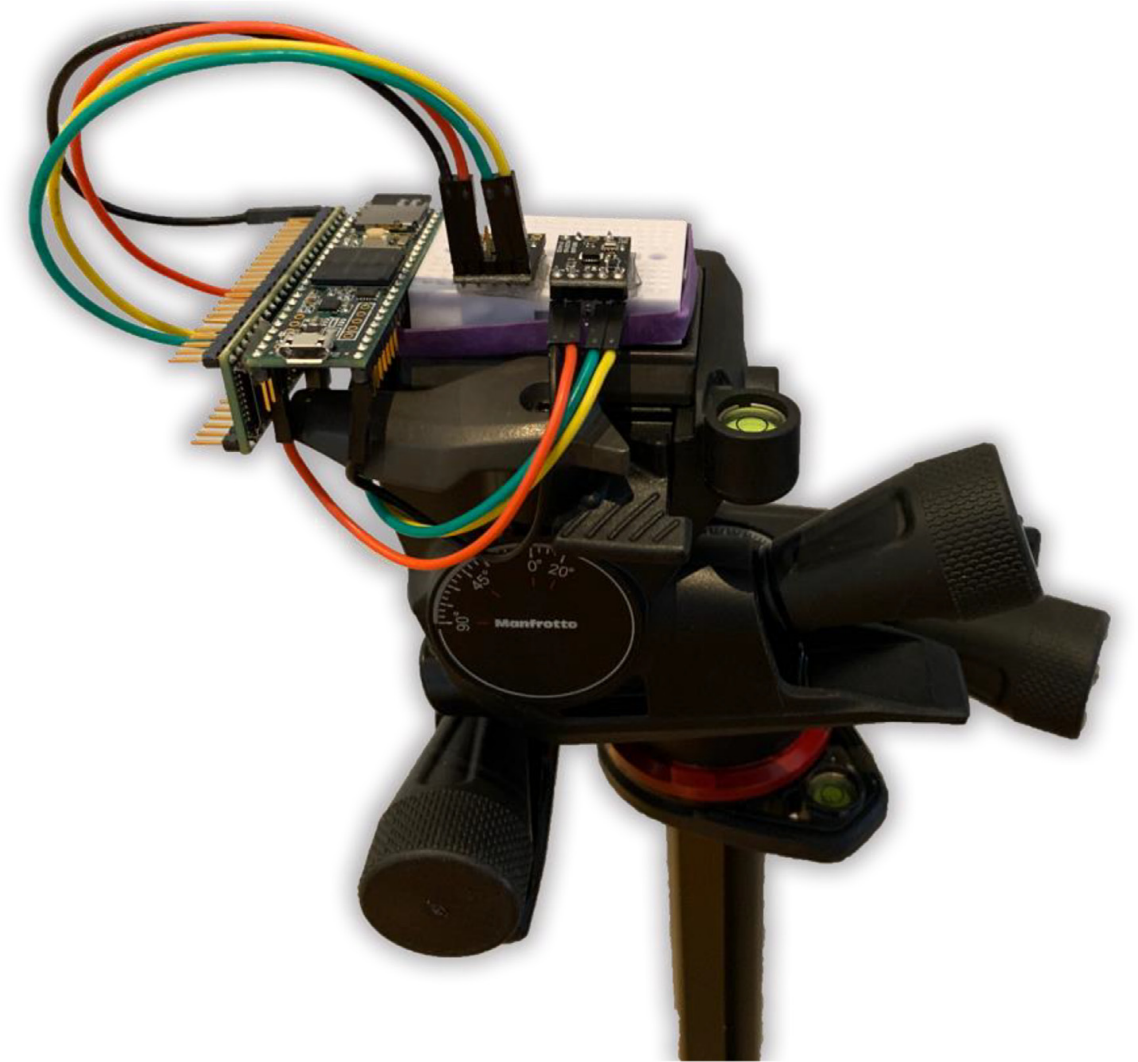
**IMU Sensor Validation Setup.** Shown is a geared tripod head on which the two IMU sensors were simultaneously validated.

Concurrent validity was established using a geared tripod head (Manfrotto MHX-PRO, Lino Manfrotto + Co. Spa). Concurrent validity is demonstrated when a test correlates well with a measure that has previously been validated.[11] In this case, the test was the positional orientation reported by the IMU sensor and the measure was the position of the geared tripod head. A geared tripod head is a tripod head that allows for precise adjustment in 3 perpendicular axes. Each individual axis is controlled by a knob and the position can be tuned precisely using a protractor-like scale. Two IMU sensors that were rigidly attached to the center of the geared tripod head for simultaneous validation testing (Fig. 4).

The measurable range of the geared tripod head was −20 to 90 degrees for the pitch and roll axes and −180 to 180 degrees for the yaw axis. Each 5-degree marking was considered a ‘stop’ for validation testing. This amounted to a total of 23 stops each for the pitch and roll axes and 73 stops for the yaw axis. A random number generator was used to determine the order of the validation sequence. For each testing position, the geared tripod head was adjusted to the specified position. Once positioned, a keypress initiates datalogging on the sensor. Three seconds (30 values at 10hz) were averaged to represent the final sensor reading which would then be compared to the actual position on the geared tripod head. Between each validation stop, the geared tripod was returned to a zeroed state.

**Fig. 5:**
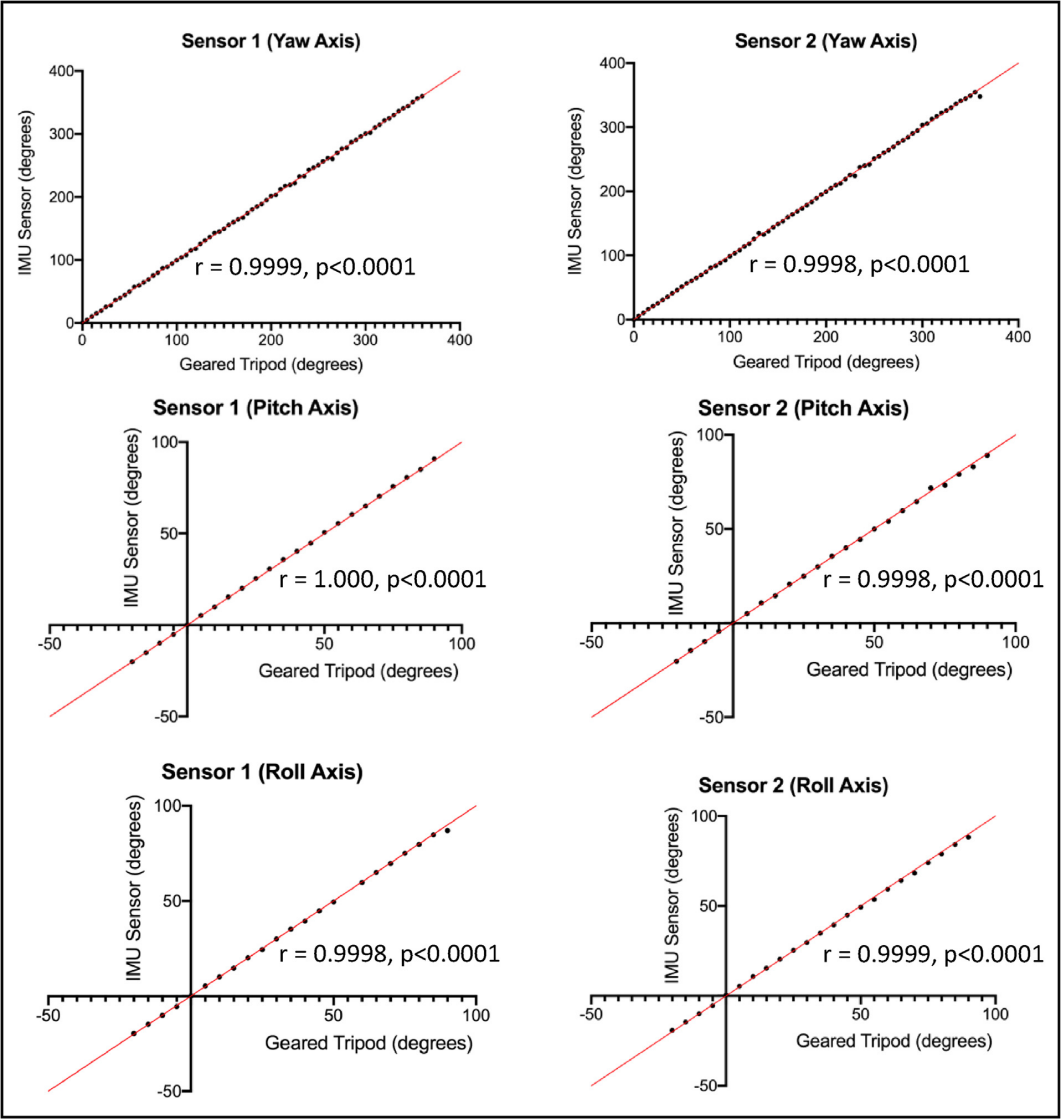
**Pearson Correlation Coefficients.** Correlation between positional orientation measurements by the geared tripod head (x-axis) and the IMU sensor (y-axis). Plots were constructed for each of the yaw, pitch, and roll axes for both IMU sensors. Statistical significance for the correlation coefficient was set at p < 0.05. The correlation coefficient is shown overlaid for each comparison.

**Fig. 6:**
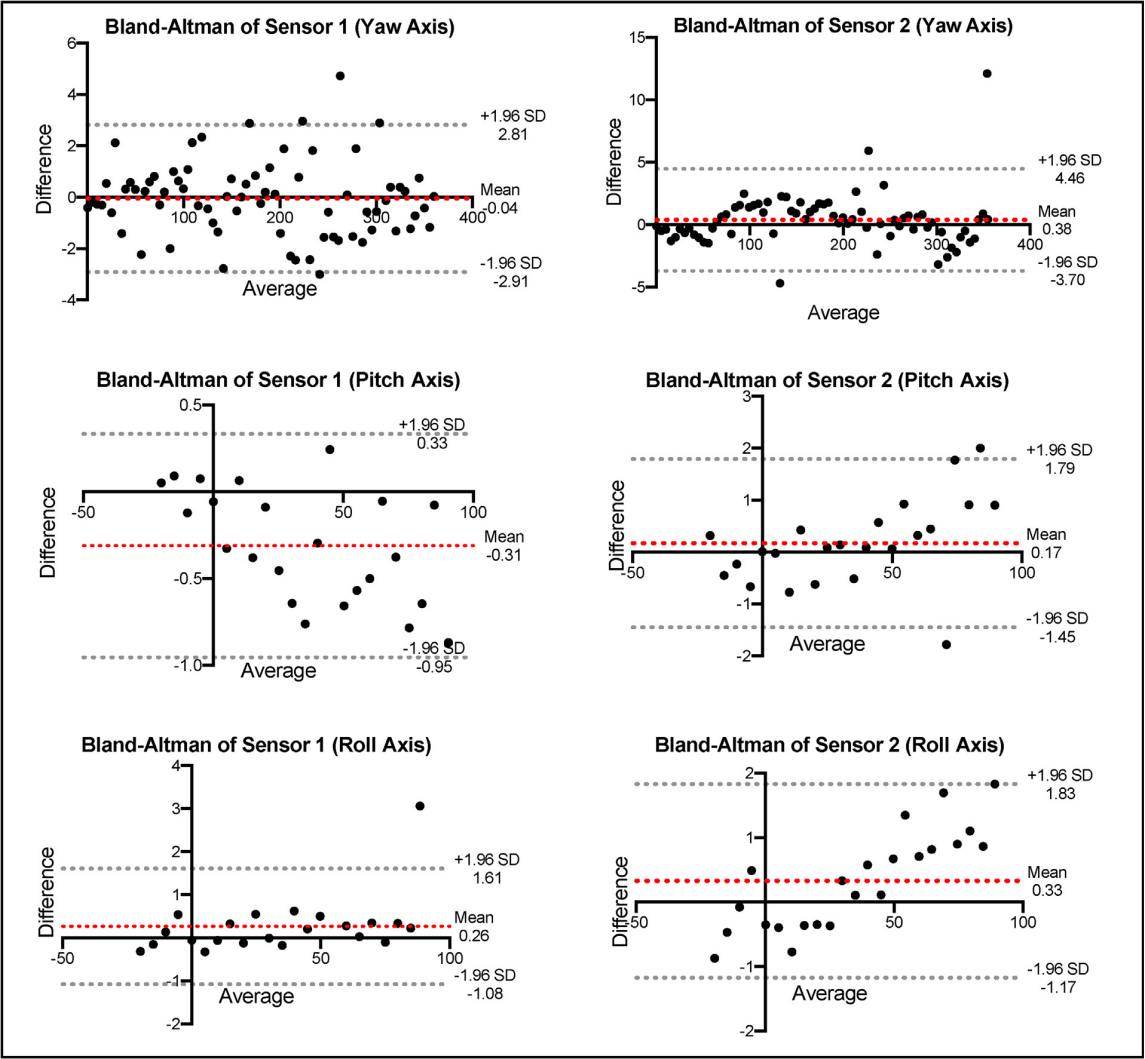
**Bland-Altman Plots for IMU Sensor Validation.** Shown are the Bland-Altman plots comparing IMU sensor measurement to the geared tripod head position. 95% limits of agreement are shown in each plot as a grey dotted line and the mean of the differences is shown as the red dotted line. (For interpretation of the references to colour in this figure legend, the reader is referred to the web version of this article.)

Agreement between the two measurement techniques was assessed using Pearson’s correlation coefficient and also by Bland-Altman plot analysis. Pearson’s correlation coefficient alone is not adequate for establishing concurrent validity because the technique doesn’t account for systemic biases that may be present in measurements and therefore Bland-Altman plots are the preferred method of evaluating agreement between two measurement techniques.[12], [13] Bland-Altman plots quantify agreement between two quantitative measurements by constructing limits of agreement, which are calculated by using the mean and the standard deviation of the differences between two measurements.[14] Fig. 5 shows agreement as assessed by the Pearson correlation coefficient and Fig. 6 shows the Bland-Altman plots for the same comparison.

Overall, the sensors showed excellent agreement with the geared tripod head as shown both in the Pearson correlation coefficient analysis as well as the Bland-Altman analysis. The Pearson correlation coefficient for each comparison was very high (near one) with strong statistical significance (p < 0.001). The Bland-Altman plots demonstrate no significant systemic bias in measurement with limits of agreement acceptable for use in this study. Concurrent validity has been established for the IMU sensors, and they can now be reliably used for measuring positional orientation.

## 8 Characterization

As described earlier, our research group has developed a novel wearable camera for intraoperative video recording of surgical procedures. This is a shoulder-mounted, motorized, gimbal-stabilized camera device that also harnesses computer vision-based object tracking that has been designed specifically to address many of the limitations of currently available camera systems. As part of the development of this device, we sought to objectively compare our prototype device to the head-mounted GoPro, as this was identified to be the most commonly used device and configuration for this type of video capture. One of the performance metrics deemed important was camera movement throughout a surgical procedure.

An experimental design was developed for objective comparison of the prototype camera system to the head-mounted GoPro. Two separate surgeons performed repeated skin procedures in a simulated operating room setting. The procedure performed was a Z-plasty transposition flap on a porcine-based skin model. [15] The surgeon was outfitted with both the head-mounted GoPro and the motorized, gimbal-stabilized, shoulder-mounted prototype camera system simultaneously. The validated IMU sensors described in this paper were affixed to each device. IMU sensor data logging as well as video capture were synchronized so that direct, time-matched comparisons could be made.

IMU sensor data was sampled at an arbitrary frequency of 10 Hz. Sensor data was in the form of quaternions and Euler angles. Euler angle displacement was calculated by taking the difference between subsequent Euler angle data points. The total angular displacement for the yaw, pitch and roll axes were calculated for each trial case. These values were then divided by the case length to generate the average Euler angular displacement, in degrees per minute, for each axis. In a similar manner, rotation quaternions were calculated between subsequent quaternion data points (Equation 3). This represents the rotation from one quaternion to the next along the shortest arc. The axis-angle of the rotation quaternion was then calculated (Equation 4). The difference in quaternion rotation angles was calculated for each subsequent data point. The total angular displacement from the rotation quaternions was then calculated for each trial case. This total value was then divided by the case length to generate the average quaternion angular displacement, in degrees per minute. The trial-specific Euler angular displacement and quaternion angular displacement were then averaged for overall values. Statistical analysis was performed. A paired two-sample *t*-test was used to compare mean angular displacement values for all of the included trials. An alpha of 0.05 was selected as the cut off for statistical significance. Results are presented as mean ± standard deviation, unless otherwise specified.

$$\mathrm{qR}=q2*{q1}^{-1};$$

where qR represents the rotation quaternion,q1, q2 represent start and end quaternions, respectively

**Equation 3:** Calculating the rotation quaternion.

$$\theta =2*\cos^{-1}\left(qR\right);$$

where θ represents the axis-angle of the qR in degrees

**Equation 4:** Calculating the exact angle of rotation.

**Fig. 7:**
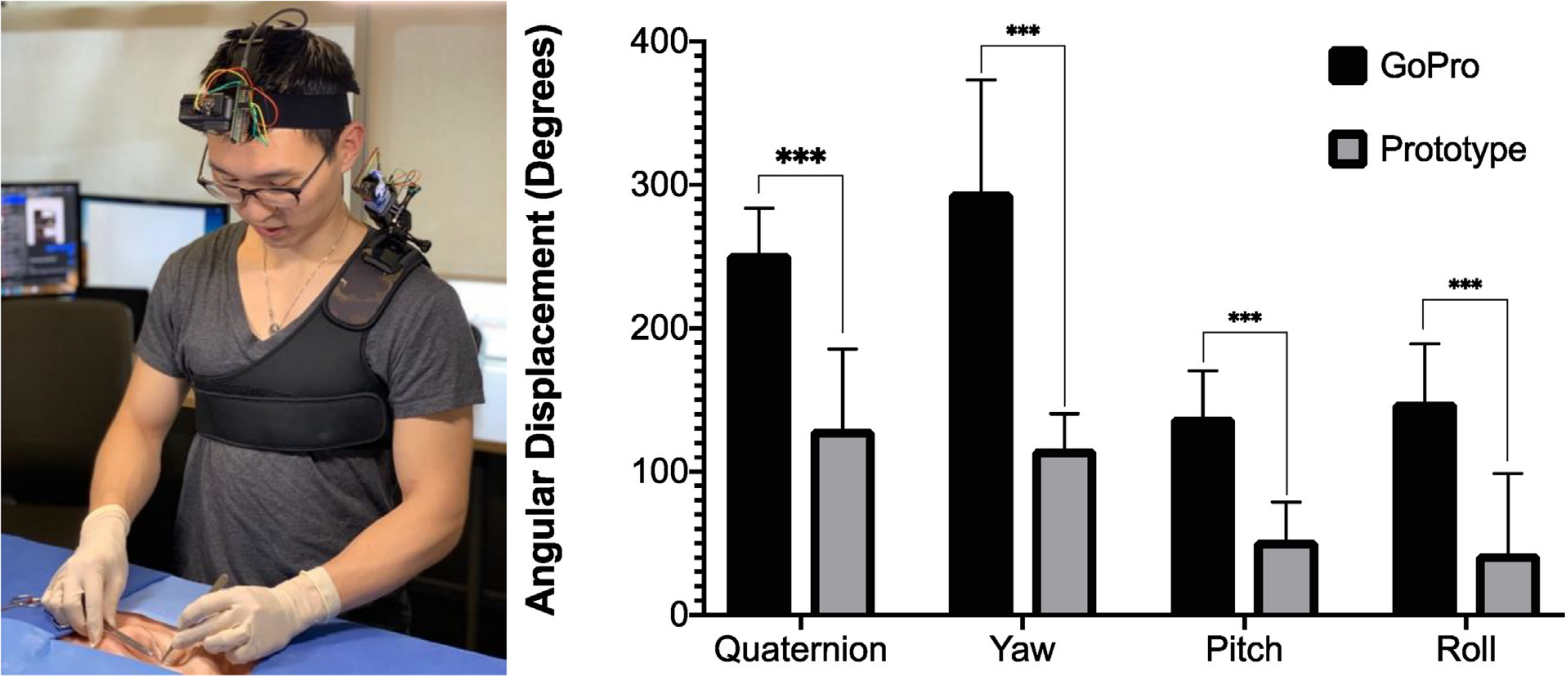
**Physical Camera Movement.** (Left) Shown is the experimental setup with both the hea-mounted camera and the motorized, gimbal-stabilized, shoulder-mounted prototype camera. (Right) The average angular displacement, normalized for case length as degrees per minute, is shown on the Y-axis. Angular displacement was calculated from rotation quaternions as well as independently for each rotational axis represented by Euler angles, shown on the X-axis.

A total of 12 skin procedures were performed by two separate surgeons, amounting to 6 procedures per surgeon. The average procedure length was 9.2 ± 1.7 min. IMU motion data was logged as described above. Video data from both cameras was captured and coded according to camera type and trial number. The gimbal-stabilized prototype camera system demonstrated significantly less movement than the head-mounted GoPro when analyzed as quaternions (130 ± 56 vs. 253 ± 31, p < 0.001) and as Euler angles (Yaw: 116 ± 58 vs. 295 ± 78, p < 0.001, Pitch: 52 ± 26 vs. 138 ± 32, p < 0.001, Roll: 43 ± 26 vs. 149 ± 40, p < 0.001) (Fig. 7). This characterization experiment, which relies on the surgical camera movement sensor described in this paper, assists in the development of a motorized, gimbal-stabilized, shoulder-mounted camera for intraoperative video-recording as it begins to provide objective evidence that this prototype system has less physical movement than the commonly used head-mounted action camera.

## 9 Conclusion

This article has presented an open source IMU-based sensor system for quantifying motion of wearable camera devices. Built from off-the-shelf components, this sensor system is an affordable, customizable, and light-weight solution compared to commercially available IMU devices. The novelty of the sensor system described is that it is compatible with current devices being used for intraoperative video capture and can reliably capture and log data within the operating room environment. The sensor system has successfully been used for its intended purpose of comparing motion between two different wearable camera devices worn by a surgeon while performing surgery. Future development will focus on increasing portability and creating lightweight shells to contain and protect the electronics.

## Declaration of Competing Interest

The authors declare that they have no known competing financial interests or personal relationships that could have appeared to influence the work reported in this paper.
